# Patient and Clinician Experience of Using Telehealth During the **'COVID-19** Pandemic in a Public Mental Health Service in Australia

**DOI:** 10.1093/schizbullopen/sgad016

**Published:** 2023-07-18

**Authors:** Lewis Robinson, Charles Parsons, Korinne Northwood, Dan Siskind, Peter McArdle

**Affiliations:** Metro South Addiction and Mental Health Services, Brisbane, QLD, Australia; Metro South Addiction and Mental Health Services, Brisbane, QLD, Australia; Metro South Addiction and Mental Health Services, Brisbane, QLD, Australia; School of Clinical Medicine, The University of Queensland, Princess Alexandra Hospital, Brisbane, QLD, Australia; Metro South Addiction and Mental Health Services, Brisbane, QLD, Australia; School of Clinical Medicine, The University of Queensland, Princess Alexandra Hospital, Brisbane, QLD, Australia; Metro South Addiction and Mental Health Services, Brisbane, QLD, Australia; School of Clinical Medicine, The University of Queensland, Princess Alexandra Hospital, Brisbane, QLD, Australia; School of Medicine, Griffith University, Brisbane, Australia

**Keywords:** Mental Health, schizophrenia, COVID-19, telehealth, telepsychiatry

## Abstract

**Background:**

During the coronavirus disease 2019 (Covid-19) pandemic, mental health services adopted telehealth to facilitate ongoing assessment and treatment of patients with severe mental illness. We aimed to assess the telehealth experience of mental health patients and clinicians during the COVID-19 pandemic to inform ongoing clinical telehealth service usage.

**Methods:**

Two participant cohorts were recruited: Patients with severe mental illness at a community public mental health service; and clinicians working within this service. Participants from both cohorts were surveyed regarding their experience of using telehealth.

**Results:**

The survey was completed by 44 patients and 51 clinicians. Most participants reported having access to appropriate telehealth technology. Among patients, 80% reported having participated in any telephone consultations, while 39% reported having taken part in video-telehealth consultations with their psychiatrist. Similarly, 77% of clinicians reported having used video telehealth. Patients reported feeling more confident with video telehealth if they were younger, lived with friends, family or partner, or had access to the internet or a smartphone. Patients reported that telehealth consultations were more convenient and may reduce nonattendance. They reported having good rapport when using video telehealth. The majority of clinicians reported feeling positively about assessing risk and delivering therapy using video telehealth but not with telephone consultations.

**Conclusions:**

Our study suggests that video telehealth is a feasible way of delivering mental health care and appears to be acceptable to both patients and clinicians. However, clinicians raised concerns about their ability to assess risk and provide therapy using telephone consultations. Patients also reported that the convenience of telehealth may improve engagement.

## Introduction

During the coronavirus disease 2019 (COVID-19) pandemic, many health services developed plans to increase the capacity for emergency care within the hospital system, as well as to limit non-essential physical contact that could contribute to the spread of COVID-19 in the community and amongst the health workforce.^1^

One such measure was to replace face-to-face appointments, where possible, with telehealth consultations.^2^ These telehealth consultations were carried out via telephone or videoconferencing platforms.^2^

At the start of the COVID-19 pandemic, telehealth consultations were adopted globally across a wide range of specialist services, including mental health.^3,4^ As the pandemic unfolded, telehealth often became the default way of delivering psychiatric outpatient care especially when social distancing measures were in place or during periods of “lock-down.”^5^ This switch to telehealth was particularly pertinent in mental health, as telepsychiatry is potentially especially well suited to the delivery of mental health care because mental health assessments primarily involve audio–visual communication and rely less on physical examination.^6^

“The public mental health care system for people with severe and persistent mental illness in Australia is delivered by State government health departments through public psychiatric hospitals and public mental health outpatient clinics, similar to the US Veterans Administration outpatient clinics.^7^ All patients with severe mental illness open to the outpatient clinic are assigned a case manager (nurse, social worker, occupational therapist, or psychologist) within a clinical outpatient team, with a psychiatrist and psychiatry trainee associated with that team. All participants in this study were outpatients of public mental health clinics. Psychotropics are subsidized through the universal health insurance system.”

During the first 2 years of the pandemic, Queensland, the state in which this study was conducted, had been successful in suppressing COVID-19 transmission in the community. This was achieved through a combination of strict public health measures including border controls, quarantine, mask-wearing, and lockdowns. During this time, a mass vaccination program was rolled out.

In mid-December 2021, many restrictions were lifted including the removal of travel restrictions between Queensland and other Australian states and removal of mandatory quarantine for some international travelers. Shortly after this, Queensland experienced its first waves of widespread COVID-19 transmission in the community. Many COVID restrictions remained in place during this period including the requirement for mask-wearing in health settings and on public transport, and the recommendation to avoid face-to-face clinical assessments where possible, in favor of telehealth.

Despite the widespread implementation of telehealth during the pandemic, effectiveness and implementation studies examining telehealth-delivered mental health care are limited.^8–10^ It has been suggested that the use of telehealth to engage people living with mental illness may be feasible, acceptable, and effective.^11^ Studies have also suggested that telehealth in psychiatry may have similar clinical efficacy to in-person care for a range of high prevalence mental health conditions including depression, anxiety, PTSD, OCD, and adjustment disorder.^12,13^

There is some debate in the literature about preferences for video vs telephone-delivered telehealth. Some studies have suggested that clinicians find building rapport, assessing risk, mood, and affect may be more challenging using telehealth compared to face-to-face interviews, especially if video telehealth is not available.^14^ Conversely, a recent analysis of telehealth use in a regional mental health service suggested that staff and patients preferred the use of telephones over videoconferencing technology, citing limitations due to infrastructure and privacy concerns.^15^

The acceptability of telehealth amongst different sub-populations with mental illness who may have different levels of experience with technology, notably people with schizophrenia, is also not clear.^16^ Some have argued that the use of telehealth in patients with psychotic illnesses such as schizophrenia may be more problematic due to delusional beliefs about technology or technophobia.^12^

In addition to patient-related factors, a recent review of barriers to the use of telehealth also found that clinicians’ concerns represented one of the main obstacles to the broader implementation of telehealth technologies.^17^ It has been suggested that younger, more tech-savvy clinicians may be more accepting of the use of telehealth.^18^

To help guide understanding of the feasibility and acceptability of tele-mental health services among people with severe mental illnesses including schizophrenia, we examined staff and patient exposure to telehealth in a metropolitan public mental health service in Brisbane, Australia during an active phase of the COVID-19 pandemic. We also assessed satisfaction with telehealth as a modality for providing and receiving mental health care, and explored whether technical issues were experienced. This article reports the findings of a survey conducted between March and July 2022 to evaluate clinician and patient experience of using telehealth-delivered mental health care in a metropolitan public mental health service during these first waves of the COVID-19 pandemic.

## Methods

Ethical approval for this study was granted by the Metro South Human Research Ethics Committee (HREC/2021/QMS/79203).

Participants were recruited from 2 cohorts. The first were patients with severe mental illness attending the mental health outpatient department of a large metropolitan mental health service in Queensland. The second were mental health clinicians and medical staff working in this same department. Data were collected from March to July 2022, during a period of high levels of COVID transmission in this region, and a clinical service recommendation to avoid of face-to-face clinical appointments where possible. Participation was undertaken on a voluntary basis, and informed consent was sought prior to participation in the study.

Data were collected using separate patient and clinician surveys (available in supplementary items). Survey responses were collected anonymously and were not re- identifiable. Data collected included demographic information including age, sex, and indigenous status, as well as Likert scales for participants to self-report their experience of and attitudes towards using telehealth-delivered mental health care and information on technical issues encountered during the use of this technology.

Data analysis were performed in the R environment.^19^ The sample population was summarized using descriptive statistics. Inferential analysis included Chi-squared analysis of proportions, ANOVA for multiple measures, and Kruskall–Wallis test statistic for ranked ordinals. Pearson correlations were performed to explore relationships between variables.

## Results

A total of 51 mental health clinicians and 44 patients completed the telehealth questionnaires. Of the 51 mental health staff completing the survey, 25 were medical (Psychiatrists or Psychiatry trainees) and 26 were nursing and allied health staff (including nurses, psychologists and occupational therapists, and social workers). There were equal number of male and female staff survey respondents. Nineteen staff (37.3%) were aged 18–34, 25 (49.1%) were aged 35–54, and 5 (9.8%) were over 55, with 2 not providing their age.

Sixty-two patients were invited to participate in the survey. Of these, 44 patients (71%) completed the questionnaire. Of the 44 patients who completed the survey, 36 had a primary diagnosis of schizophrenia, 4 had a mood disorder, 2 had a personality disorder, and the remaining 2 had been diagnosed with an eating disorder. Eighteen (42.9%) were aged 18–34, 20 (47.6%) were aged 35–54, and 4 (9.5%) were over 55. Two (4.6%) identified as indigenous and 70% were unemployed. The demographic characteristics of patients are displayed in table 1.

**Table 1.** Characteristics of Patients (*n* = 44)

Diagnosis	Consumers *n*(%)
Schizophrenia	36
Mood disorder	4
Personality disorder	2
Eating disorder	2
*Age*	
18–34	18 (42.9)
35–54	20 (47.6)
55+	4 (9.5)
*Gender*	
Woman	22 (50)
Man	21 (47.7)
Non binary	1 (2.3)
*First nations*	
ATSI	2 (4.6)
No	42 (95.5)
*Employment*	
Student	6 (13.6)
Employed	7 (15.9)
Unemployed	31 (70.5)
*Living situation*	
With others	33 (75)
Boarding house/hostel	3 (6.8)
Supported	2 (4.6)
Alone	6 (13.6)
*Transport*	
Private vehicle	25 (56.8)
Public transport	9 (20.5)
Supported by worker	5 (11.4)
Rideshare	2 (4.6)
Walking	3 (6.8)
*Travel time*	
<10 min	7 (15.9)
10–30 min	31 (70.5)
30–60 min	6 (13.6)

### Current Telehealth Activities

Most patients reported having access to technology for telehealth, with 95% of patients having telephone access (either smartphone, non-smartphone, or landline telephone) which they reported using “once to several times per day.” Almost all (91%) patients reported having access to video capability, either via smartphones, and/or computer with webcam; 70% of patients reported having access to internet or mobile data capabilities to engage in telehealth, and nearly all of these (67.7% home internet, 70.2% mobile data) have greater than 5 GBs of data available for use.

Patients reported that they were more likely to have had phone rather than video consults with their treating team. Most (80%) patients reported that they had participated in telephone consultations with their psychiatrist and 64% reported experience of telephone reviews conducted by their case manager. Less than half (39%) of patients reported having had exposure to video consultations with their psychiatrist and 27% reported having used video telehealth with their case manager.

The majority of clinicians (77%) reported having experience with using video telehealth for therapeutic work but only 22% had used video telehealth in the past week. Most clinicians reported great or easy access to video telehealth technology (65%), with a minority reporting limited, difficult, or no access (20%). Clinician access to video telehealth did not differ significantly based on age or gender.

### Patient Confidence With Video Telehealth

Patients were significantly more confident with video telehealth if they were younger (*P* = .015), lived with family, friends, or a romantic partner (*P* = .033), had access to a smartphone (*P* < .001) and had access to home internet (*P* = .020). Patients who are older, lived alone or in supported accommodation (boarding house, hostel, and medium to high care facility) or did not have access to a smartphone or home internet were significantly less confident in the use of video telehealth. Employment status, gender, and ATSI cultural identity did not significantly impact on confidence with video consultations.

### Patient Impressions of Phone and Video Telehealth

**Fig. 1.**
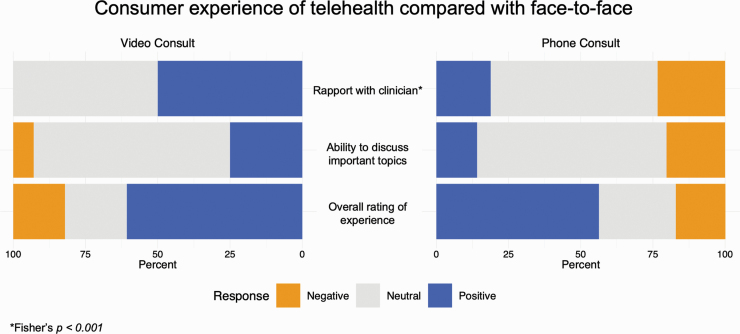
Consumer experience of telehealth compared with face to face. N.B. Consumer is the preferred term for patient in the clinical service where this study was undertaken.

Patients reported that they developed better rapport with their clinicians using video telehealth but not telephone reviews when each modality was compared to traditional face-to-face appointments (*P* < .001) (figure 1). Patients reported that their ability to discuss important topics was not hampered by the use of telehealth compared to face-to-face care. The patients experience of using telehealth did not appear to differ significantly between consultations with their doctor and their case manager (figure 1).

One-third (34%) of patients reported that they had previously missed a face-to-face appointment with their psychiatrist. The majority of patients reported that telehealth appointments were more convenient (82%) and 59% felt that the availability of this option would make them less likely to miss an appointment in future.

### Clinician Experience of Telehealth Compared With Face-to-Face

**Fig. 2.**
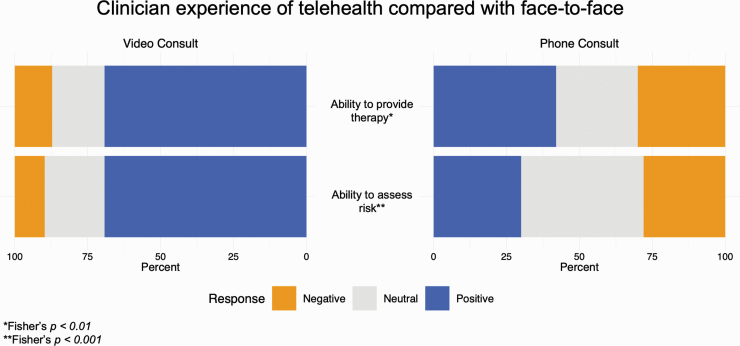
Clinican experience of telehealth compared with face to face.

The majority of clinicians (62.9%) felt positively about their ability to provide therapy using video telehealth. However, the majority of clinicians (58%) were unsure or felt negatively about the quality of clinical care delivered via telephone consultations. Most clinicians (69.2%) reported feeling comfortable assessing risk using video telehealth, while only 30% of clinicians felt positively regarding their ability to accurately assess risk during a telephone review (figure 2). The majority (88%) of clinicians felt that having a telehealth option has a positive effect on patients’ willingness to engage with the service.

### Interest in Telehealth

Clinicians were significantly more interested in using video consultations as a way of providing mental health care than patients (*P* < .001). Patients interest in video consultations with their case manager was significantly lower than interest in video consultations with their doctor (*P* < .001). Patient interest in telehealth consultations did not appear to be significantly affected by their confidence in using the technology or their access to a smartphone.

## Discussion

This article reports the findings of a survey conducted between March and July 2022 to evaluate clinician and patient experience of using telehealth-delivered mental health care in a metropolitan public mental health service during the first waves of the COVID-19 pandemic in Queensland. Telehealth was particularly relevant during this period, with government recommendations to use telehealth instead of face-to-face clinical assessments if possible.

Patients who participated in the study were significantly more likely to rate video consultations as having a positive impact on rapport with their clinicians than consultations delivered by phone when compared to face-to-face consultations. Patients reported feeling more able to discuss important topics in consultations delivered through video telehealth than by phone, though this difference did not reach statistical significance. Most patients reported they were satisfied with the overall experience of both phone and video consultations.

Similarly, clinicians generally reported a positive experience of using video telehealth to provide mental health care. In particular, the majority of clinicians felt positively about their ability to assess risk and conduct therapeutic work with their patients during video consults. In contrast, many clinicians had doubts about their ability to adequately assess risk and provide therapeutic work during telephone consultations. The finding of an improved ability for patients and clinicians to build rapport using video telehealth is in keeping with previously published work.^12^

Despite the majority of clinicians reporting great or easy access to video telehealth, phone consultations were the most frequently used telehealth modality in the study population. It is also interesting that one-fifth of clinicians reported difficult or no access to video telehealth, despite all clinicians having theoretical access to the same technological infrastructure. This suggests that there may be other difficulties in utilizing video telehealth technology with patients in a public metropolitan mental health service. Previous studies have highlighted some possible challenges including inadequate infrastructure and privacy concerns.^13^ There may be other constraints such as lack of training as well as the additional time and organization required to set up video consultations compared to those carried out by telephone.

Patients who were older, lived alone or in supported accommodation (boarding house, hostel, and medium to high care facility), or did not have access to a smartphone or home internet were significantly less confident in using video telehealth technology. Employment status, gender, and indigenous identity did not significantly impact on patient’s confidence with video consults. Patients were significantly more confident with video telehealth if they are younger, lived with family, friends or a romantic partner, and had access to a smartphone or home internet.

The majority of clinicians reported high levels of interest in continuing to provide video telehealth services. This finding is in keeping with prior research on youth mental health services.^7^ In contrast, patients participating in this study were significantly less interested in video telehealth services than clinicians. In addition, patients were significantly less interested in telehealth consultations with their case manager than with their psychiatrist or registrar.

Given the reports of a positive experience with telehealth, the relative lack of patient interest in using telehealth in the future was unexpected. It is possible that this reflects some patients’ overall reluctance to engage with mental health services. Subgroup analysis did not demonstrate a significant difference in interest resulting from confidence with or access to technology.

The majority of patients reported that telehealth (phone or video) was more convenient than attending for a clinic appointment. Prioritization of convenience over the quality of the telehealth interaction may explain why patients report no overall difference in satisfaction between video or phone consultations despite patients feeling that they develop better rapport with clinicians during video consultations. Concerns have also been raised by some researchers that patients may favor telehealth over face-to-face consultations as way of avoiding direct contact with mental health services.^7^

Disruptive technical difficulties during video conferencing were reported by very few patients and were comparable to prior studies in regional areas.^13^ Privacy concerns around telehealth appointments were reported by a small minority of patients. It is possible that these concerns may be more prominent in specific groups of patients such as those with a history of psychosis.^12^

This study has several important limitations. The study examines a small sample of patients and mental health clinicians from one metropolitan mental health service in Australia. As such, the findings may not be generalizable to other mental health services. However, while we acknowledge the small sample size, we believe that the patient cohort who participated in this research is generally representative of patients who are looked after by the public mental health systems in Australia and in other jurisdictions with universal health care.

Furthermore, where concerns have been raised about patients’ ability to engage with telehealth, most concerns have been raised about those patients with severe mental illness. We believe that if the patients in our study, predominantly those with psychotic illness, can access, utilize and express positive views about telehealth-delivered mental health care, this may reflect the situation in the wider psychiatric patient population.

Further limitations in the study include the number of patients within the survey who had experience with video telehealth consultations was limited (one-third with a psychiatrist, one quarter with a case manager). It is also possible that there may also be a risk of sampling bias, with patients who were more confident in using technology perhaps being more likely to participate in the survey.

This study suggests that despite the widespread adoption of telehealth during the COVID-19 pandemic, high levels of clinician access to video health technology and an interest in delivering video consults, phone calls remain the most commonly used telehealth modality.

This study reports potential advantages in the use of video compared to phone telehealth in terms of developing patient rapport, clinician risk assessment, and therapeutic work. Patients valued the convenience of telehealth but have significantly less interest in utilizing video consults than their clinicians.

A number of studies have examined the adoption of telehealth as a way of continuing provision of mental health care during the COVID-19 pandemic. However, most studies have looked at either the experience of the patients or the alternatively the experience of clinicians working within a mental health service. The current study investigates both the patient and clinician experience and access to telehealth-delivered mental health care in a single mental health service.

Previously, some authors have raised concerns that patients with severe mental illness may struggle with telehealth due to poor computer literacy and limited access to the internet. The current study suggests that patients with severe mental illness in Australia generally have access to technology capable of supporting telehealth consultations and are able to utilize this technology successfully.

As COVID enters an endemic phase, services will have to adjust the delivery of mental health care in a new “COVID normal” environment. Our study suggests that video consults are acceptable to patients and clinicians alike and should remain an option for delivering mental health care as we move forward from the pandemic. Telephone consultations appear to have less utility due to difficulties in developing rapport and assessing risk. Patient preference for the convenience of telephone-delivered mental health care should be balanced against the risks of delivering suboptimal care quality and facilitating avoidance.

A return to face-to-face care should be prioritized for patients who may struggle with the use of technology such as those who are older, live alone or in supported accommodation, or don’t have access to a smartphone or home internet as they are significantly less likely to be confident using in video telehealth technology.

## Supplementary Material

sgad016_suppl_Supplementary_Data
