# Supplementary material for: Patient and Clinician Experience of Using Telehealth During the 'COVID-19 Pandemic in a Public Mental Health Service in Australia
Source: Schizophr Bull Open. 2023 Jul 18;4(1):sgad016. doi: 10.1093/schizbullopen/sgad016 (PMC11441319; doi:10.1093/schizbullopen/sgad016)
Supplement: sgad016_suppl_Supplementary_Data [file sgad016_suppl_Supplementary_Data.docx]

**Supplementary Item 1 – Consumer Survey.**

**Demographics and screening**

How old are you?

Gender Identity

- - Woman
  - Man
  - Non Binary/Gender Fluid
  - X
  - Other (Please specify
  - Prefer not to say

Are you of Aboriginal or Torres Strait Islander background?

- - No
  - Aboriginal
  - Torres Strait Islander
  - Aboriginal and Torres Strait Islander
  - Prefer not to say

Which of the following best describes your employment situation status?

- - Full time student
  - Part time student
  - Full-time worker in paid employment
  - Part-time worker in paid employment
  - Unpaid worker as a parent or carer
  - Currently unemployed (Disability Support Pension)
  - Currently unemployed (Centrelink Benefits).
  - Currently unemployed (privately funded - no government benefits).

What is your primary mode of transport to attend appointments?

- - Private car (driver)
  - Public transport.
  - Taxi/Rideshare (Uber)
  - Support Workers.
  - Walking.
  - Motorbike.
  - Bike/Scooter (or other form of personal non licensed transport)

How long does it usually take you to travel to your mental health appointments?

- <10 minutes
- 10-30 minutes
- 30-60 minutes
- 1-2 hours.
- >2 hours

What is your living situation currently?

- - Living with family/friends/romantic partner
  - Living in shared accommodation (boarding house/hostel), including with medication, meal and laundry support.
  - Living in medium to high care supported accommodation including nursing home, SIL (supported independent living), SDA (Supported Disability Accommodation).
  - Living alone
  - Homeless or couch surfing

Do you require a mobility aid (wheelchair, four-wheel-walker, walking stick) to attend the clinic?

Yes/No

Do you require a support worker for your attendance at the clinic?

Yes/No

**Technology ownership and access**

How would you rate your level of confidence in using technology?

1 = very unconfident,

2 = unconfident,

3 = neutral,

4 = confident

5 = very confident

Do you personally own or have private access to a smartphone (a mobile device that can run apps and connect to the internet)?

- - If yes, then:
    1. How often do you use this technology?
       1. Several times an hour
       2. Once an hour
       3. Once to several times a day
       4. Once to several times a week
       5. Less than once a week
    2. How much mobile internet data do you have access to each month?

Less than 2 GBs, 2 to 5GBs, more than 5GB's, Not sure

- - Don’t know

Do you personally own or have private access to a non-smartphone (a mobile device that can make calls and send texts, but can’t run apps or connect to the internet)?

- 1. If yes, then:
     1. How often do you use this technology?
        1. Several times an hour
        2. Once an hour
        3. Once to several times a day
        4. Once to several times a week
        5. Less than once a week
  2. No
  3. Don’t know

Do you have a Tablet, Laptop or desktop computer?

- 1. If yes, then:
     1. How often do you use this technology? (single select)
        1. Several times an hour
        2. Every hour/once an hour
        3. Once to several times a day
        4. Once to several times a week
        5. Less than once a week
  2. No
  3. Don’t know

If yes, does this device have access to webcam (video) capabilities?

Do you have access to wi-fi at your primary place of residence?

Yes/no

If yes, how much data do you have access to each month?

- 1. Less than 2GBs, 2 to 5GBs, more than 5GB's, Not sure

**Exposure to Telehealth**

How often do you have contact with your **Psychiatrist or Registrar**?

(Once a week or more, once per month or more, less than once per month)

Have you had a consultation with your **Psychiatrist or Registrar via phone?**

Yes/No

How would you rate the experience of having a consultation by phone compared to seeing your doctor in person?

Very satisfied, satisfied, neither satisfied nor dissatisfied, dissatisfied, very dissatisfied

How did talking via the phone impact your ability to discuss the topics that are important to you?

Much improved, Improved, no change, worse, much worse.

How do you feel having a consultation over the phone impacted your rapport with your Psychiatrist or Registrar?

Much improved, Improved, neutral, worse, much worse.

Did you experience any technical difficulties?

Yes/No

If yes: Very often, often, not often, rarely, not at all.

Have you had a consultation with your **Psychiatrist or Registrar via a video link?**

Yes/No

How would you rate the experience of having a consultation by video link compared to seeing your doctor in person?

Very satisfied, satisfied, neither satisfied nor dissatisfied, dissatisfied, very dissatisfied

How did talking via video link impact your ability to discuss the topics that are important to you?

Much improved, Improved, no change, worse, much worse.

How do you feel having a consultation over a video link impacted your rapport with your Psychiatrist or Registrar?

Much improved, improved, neutral, worse, much worse.

Did you experience technical difficulties?

Yes/No

If yes: Very often, often, not often, rarely, not at all.

How often do you have contact with your **Case Manager**?

(Once a week or more, once per month or more, less than once per month)

Have you had a consultation with your **Case manager via phone?**

Yes/No

How would you rate the experience of having a consultation by phone compared to seeing your case manager in person?

Very satisfied, satisfied, neither satisfied nor dissatisfied, dissatisfied, very dissatisfied

How did talking via the phone impact your ability to discuss the topics that are important to you?

Much improved, Improved, no change, worse, much worse.

How do you feel having a consultation by phone impacted your rapport with your case manager?

Much improved, Improved, neutral, worse, much worse.

Did you experience technical difficulties?

Yes/No

If yes: Very often, often, not often, rarely, not at all.

Have you had a consultation with your **Case manager via a video link?**

Yes/No

How would you rate the experience of having a consultation by video link compared to seeing your case manager in person?

Very satisfied, satisfied, neither satisfied nor dissatisfied, dissatisfied, very dissatisfied

How did talking on a video link impact your ability to discuss the topics that are important to you?

Much improved, improved, no change, worse, much worse.

How do you feel having a consultation by a video link impacted your rapport with your case manager?

Much improved, Improved, neutral, worse, much worse.

Did you experience technical difficulties?

Yes/No

If yes: Very often, often, not often, rarely, not at all.

Have you missed a scheduled appointment with your psychiatrist or registrar?

Yes/No

Would having a Telehealth consultation option (either by phone or video-link), make you less likely to miss an appointment in the future?

Yes/No

**Interest in telehealth**

What is your level of interest in using the following technologies for your mental health and wellbeing?

Using a **phone consultation** to talk to a **Doctor/Psychiatrist** about mental health and wellbeing?

Not at all interested, slightly interested, somewhat interested, moderately interested, extremely interested

Using **a consultation via video link** to talk to a **Doctor/Psychiatrist** about mental health and wellbeing?

Not at all interested, slightly interested, somewhat interested, moderately interested, extremely interested

 Using a **phone consultation** to talk to your case manager about mental health and wellbeing?

Not at all interested, slightly interested, somewhat interested, moderately interested, extremely interested

Using **a consultation via video link** to talk to your case manager about mental health and wellbeing?

Not at all interested, slightly interested, somewhat interested, moderately interested, extremely interested

If using telehealth in the future, how much do concerns for privacy worry you?
Not at all, not much, neutral, a little, very much.

How would the telehealth option impact the convenience of attending a mental health appointment, compared to attending in person?

Much less convenient, less convenient, no change, more convenient, much more convenient.

**Supplementary Item 2 – Staff Survey.**

Where do you work?

- - Early Psychosis
  - Community Team (North, East, West, South).
  - Mobile Intensive Rehabilitation Team
  - Transcultural Team
  - Other, please specify

How old are you?

- 29 or younger
- 30-39
- 40-49
- 50-59
- 60-69
- 70 or older

Which gender do you identify with?

- - Woman
  - Man
  - Non Binary/Gender Fluid
  - X
  - Other (Please specify)
  - Prefer not to say

What is your clinical role?

- - Psychiatrist
  - Psychiatry Registrar
  - Case Manager (including background of Social Work, Psychology, Occupational Therapist, Nursing).

In the last week, how many consumers have you tried to assess/review?

- - 0
  - 1-5
  - 6-10
  - 11-15
  - >15
  - Unknown

In the last week how many telephone reviews (no video) have you done?

- - 0
  - 1-5
  - 6-10
  - 11-15
  - >15
  - Unknown

In the last week how many telehealth reviews (with video) have you done?

- - 0
  - 1-5
  - 6-10
  - 11-15
  - >15
  - Unknown

In the last week, how many consumers 'failed to attend' their scheduled appointments?

- - 0
  - 1-5
  - 6-10
  - 11-15
  - >15
  - Unknown

**VIDEO TELEHEALTH**

What is your ease of access to video telehealth within your current work environment?

(No access, very limited access, difficult or time consuming to access, some access, easy access, great access).

Have you used Telehealth (with video) to clinically review and provide therapeutic care to consumers?

(yes/no)

If yes;

On telehealth (with video) how well do you feel you are able to assess risk?

- - Very well
  - Well
  - Hard to tell
  - Poorly
  - Very poorly

On telehealth (with video), how well do you feel you are able to provide clinical therapeutic work?

- - Very well
  - Well
  - Hard to tell
  - Poorly
  - Very poorly

Do you have ready access to a suitable device provided by your employer for telehealth (with video)?

Yes/No/Unsure

Would you be interested in using your own personal computer, tablet or phone device in your workplace for telehealth (with video)?

Yes/No/Unsure

**PHONE TELEHEALTH**

Have you used phone calls (no video) to clinically review and provide therapeutic care to consumers?

(yes/no)

If yes;

On phone call (no video) how well do you feel you are able to assess risk?

- - Very well
  - Well
  - Hard to tell
  - Poorly
  - Very poorly

On phone telehealth (no video) how well do you feel you are able to provide clinical therapeutic work adequately?

- - Very well
  - Well
  - Hard to tell
  - Poorly
  - Very poorly

Do you have ready access to a suitable device for phone calls (no video) provided by your employer?

Yes/No/Unsure

Would you be interested in using your own personal phone device in your workplace for telehealth (with video)?

Yes/No/Unsure

**Impact and Interest**

What impact does having telehealth (telephone or video) available have on your consumers' willingness to engage with the mental health service?

- - Very negative
  - Negative
  - Somewhat negative
  - None at all.
  - Somewhat positive
  - Positive
  - Very positive

How interested are you in using telehealth (video) to deliver mental health care to your consumers?

- - Not at all interested
  - Slightly interested
  - Somewhat interested
  - Moderately interested
  - Extremely interested
